# How residents and interns utilise and perceive the personal digital assistant and UpToDate

**DOI:** 10.1186/1472-6920-8-39

**Published:** 2008-07-14

**Authors:** Jason Phua, Tow Keang Lim

**Affiliations:** 1 Division of Respiratory and Critical Care Medicine, Department of Medicine, National University Hospital, 5 Lower Kent Ridge Road, 119074, Singapore; 2 Department of Medicine, Yong Loo Lin School of Medicine, National University of Singapore, 5 Lower Kent Ridge Road, 119074, Singapore

## Abstract

**Background:**

In this era of evidence-based medicine, doctors are increasingly using information technology to acquire medical knowledge. This study evaluates how residents and interns utilise and perceive the personal digital assistant (PDA) and the online resource UpToDate.

**Methods:**

This is a questionnaire survey of all residents and interns in a tertiary teaching hospital.

**Results:**

Out of 168 doctors, 134 (79.8%) responded to the questionnaire. Only 54 doctors (40.3%) owned a PDA. Although these owners perceived that the PDA was most useful for providing drug information, followed by medical references, scheduling and medical calculators, the majority of them did not actually have medical software applications downloaded on their PDAs. The greatest concerns highlighted for the PDA were the fear of loss and breakage, and the preference for working with desktop computers and paper. Meanwhile, only 76 doctors (56.7%) used UpToDate, even though the hospital had an institutional subscription for it. Although 93.4% of these users would recommend UpToDate to a colleague, only 57.9% stated that the use of UpToDate had led to a change in their management of patients.

**Conclusion:**

Although UpToDate and various PDA software applications were deemed useful by some of the residents and interns in our study, both digital tools were under-utilised. More should be done to facilitate the use of medical software applications on PDAs, to promote awareness of tools for evidence-based medicine such as UpToDate, and to facilitate the application of evidence-based medicine in daily clinical practice.

## Background

Evidence-based medicine involves the use of available evidence from the medical literature to optimise patient care. In recent years, physicians in training have increasingly relied on electronic resources to retrieve the necessary medical evidence to aid in their daily clinical practice [1-8]. Specifically, two digital tools – which are conceptually very different – have become especially popular. The first tool, the personal digital assistant (PDA), is a handheld computer onto which medical software applications may be downloaded, which by virtue of its unique portability can be relied upon at anytime to provide the necessary medical information at the bedside. In addition, it contains multiple functions which assist doctors in their administrative duties and personal affairs [9,10]. The second tool, the resource UpToDate, is an online tool which provides quick and pragmatic clinical information for doctors and which, though sometimes accessible on the PDA, is more often accessed on multiple computers in hospitals, clinics or homes [1,11-13].

In July 2004, we conducted a questionnaire survey on residents and interns in our tertiary teaching hospital, National University Hospital, Singapore, to evaluate their use of various traditional and electronic medical information resources, the results of which have been published [8]. To summarise, we found that while these doctors spent the most time on traditional resources like teaching sessions and print textbooks, rating them as most useful, electronic resources – especially MEDLINE – also ranked highly. Five months prior to the questionnaire survey, in February 2004, our hospital had started an institutional subscription to UpToDate. We therefore took the opportunity to use the questionnaire survey to examine the utility of the two electronic resources – the PDA and UpToDate – for residents and interns.

## Methods

We conducted the study as part of a quality improvement programme instituted to promote the practice of evidence-based medicine. Such quality improvement surveys on educational practices are exempted from a formal ethics review by our institutional review board. Our hospital has multiple specialties, each with their own set of residents and interns and their own teaching programmes. The Singaporean education system ensures a minimum level of computer literacy for these doctors, including use of the internet and basic word processing software applications without difficulty. Doctors have easy access to multiple desktop computers in all wards as well as doctors' rooms, each with an internet connection to the UpToDate website. During the time of the study, UpToDate was the only electronic resource for which our hospital had an institutional subscription. Most of the various specialty departments had either departmental or personal subscriptions to certain general medical or subspecialty journals, but whether or not the residents and interns had access to these journals online depended on each department's head and attending physicians. None of the departments provided PDAs to the doctors for official use.

We developed a questionnaire which captured information on PDA ownership, time spent using a PDA, usefulness of a PDA for acquiring medical knowledge, the medical software applications used by PDA owners, and doctors' perceptions of the various functions and potential disadvantages of the PDA. It also captured information on who used UpToDate, time spent using UpToDate, usefulness of UpToDate for acquiring medical knowledge, and their perceptions of UpToDate and its features which they felt made it popular. We used a 5-point Likert scale to identify a range of responses (1 = strongly agree or definitely useful, 2 = agree or useful, 3 = not sure, 4 = disagree or not useful, 5 = strongly disagree or definitely not useful). Other sections in the questionnaire (see Additional file 1) evaluated the use of other information resources which have been previously reported [8]. After obtaining comments on the questionnaire's face validity from colleagues within our Department of Medicine, we tested it on a pilot sample of 10 residents from our Department – changes to the questionnaire were deemed unnecessary after the pilot survey.

In July 2004, we distributed these questionnaires to all residents and interns working in our hospital through the various department secretaries. In Singapore, residents (who are beyond their first postgraduate year) undergo 6-month rotations while interns (who are in their first postgraduate year) undergo 4-month rotations in various hospitals. Therefore, most residents who received the questionnaires were undergoing postings which spanned the period from May to October 2004, while most interns were undergoing their very first clinical posting in the period from May to August 2004. The department secretaries collected the completed questionnaires within two weeks and reminded the non-responders after two, four and six weeks.

We expressed nominal data as frequencies, ordinal data as medians (interquartile range [IQR]), and continuous data as medians (IQR) or mean ± standard deviation where appropriate. We compared groups using the chi-square test, the Mann-Whitney U test and the Wilcoxon signed rank test accordingly. A p value of less than 0.05 was considered significant, with all p values being two-sided. We used the statistical software SPSS version 11.5 (SPSS Inc., Chicago, IL, USA).

## Results

A total of 134 (103 residents and 31 interns) out of 168 (133 residents and 35 interns) doctors returned the questionnaires (79.8% response rate). Table 1 demonstrates the characteristics of the respondents. The bulk of the doctors were not enrolled in any specialty training programs, as many of them had only recently graduated from medical school. In Singapore, specialty training begins in residency (not internship). In addition, the majority of Singaporean doctors become family practitioners, and to date, family practice does not require enrolment in a residency training programme.

**Table 1:** Characteristics of doctors

Characteristics	Data*
Number	
Total	134
Male	82 (61.2)
Female	52 (38.8)
	
Age, year	28 ± 3
	
Designation	
Residents	103 (76.9)
Interns	31 (23.1)
	
Training programmes	
None	84 (62.7)
Internal medicine	15 (11.2)
Anaesthesiology	8 (6.0)
Radiology	8 (6.0)
Family medicine	5 (3.7)
Paediatrics	3 (2.2)
Emergency medicine	3 (2.2)
Ophthalmology	2 (1.5)
Otolaryngology	2 (1.5)
Surgery and orthopaedics	2 (1.5)
Obstetrics and gynaecology	1 (0.7)
Psychiatry	1 (0.7)
	
Postgraduate year status	
1	31 (23.1)
2	20 (14.9)
3	11 (8.2)
4	17 (12.7)
5	19 (14.2)
6	8 (6.0)
7	10 (7.5)
8	6 (4.5)
9	3 (2.2)
10 or more	9 (6.7)

* Data are presented as number (%) or mean ± standard deviation.

### Use of PDAs

**Figure 1:**
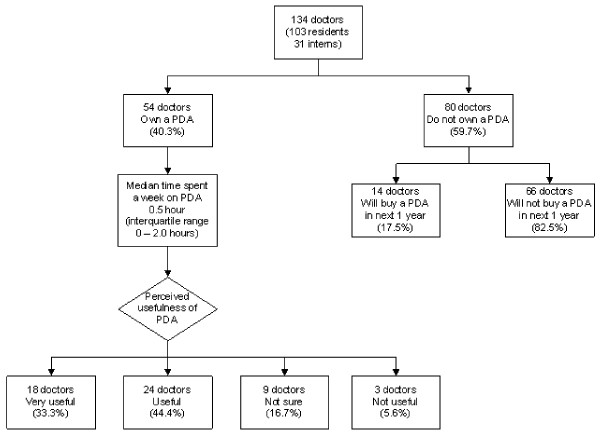
Doctors' use and perception of the personal digital assistant (PDA).

As depicted in Figure 1, 54 doctors (43 residents and 11 interns) owned a PDA. Forty-five doctors (83.3%) used a Palm operating system while 9 doctors (16.7%) used Windows CE. The majority (33 doctors, 61.1%) used their PDAs for both personal and work-related purposes, as compared to 12 (22.2%) with mainly personal purposes and 9 (16.7%) with mainly work-related purposes.

With respect to using the PDA to acquire medical knowledge, the median time spent per week was 0.5 hour (IQR 0 – 2.0 hours). The median frequency of use per week was 4.0 times (IQR 0 – 12.3 times). The median score on a Likert scale of 1 to 5 (where a score of 1 represents "definitely useful" and 5 represents "definitely not useful") for the perceived usefulness of the PDA for acquiring medical knowledge was 2.0 (IQR 1.0 – 2.0). A detailed breakdown of how these doctors rated its usefulness is provided in Figure 1.

These PDA owners perceived that the PDA was most useful for providing drug information, followed by medical references, scheduling and the calendar, medical calculators, and finally documentation (Table 2). However, in reality, half or less of these same owners had the appropriate medical software applications for drug information, medical references and medical calculators downloaded into their PDAs (Table 2). In total, the following programmes were downloaded into the 54 PDAs: drug information: the Sanford Guide to Antimicrobial Therapy (7 programmes), Epocrates (4), Physicians' Desk Reference (4), Johns Hopkins POC-IT Antibiotic Guide (2), British National Formulary (1), MIMS (1), Mosby's Drug Consult (1), Royal Children's Hospital Drug Doses (1); medical references and guidelines: 5-Minute Consult Series (26), Harrison's Manual of Medicine (12), Washington Manual of Medical Therapeutics (12), the ICU Book (4), Handbook of Evidence-based Critical Care (3), American College of Cardiology Guidelines (2), International Classification of Diseases – Ninth Revision (1), eMedicine (1), Nelson Textbook of Pediatrics (1), Schwartz's Principles of Surgery (1), Williams Obstetrics (1), Dahnert's Radiology Review Manual (1), ARTBeat (1), .911 (1); medical calculators: Archimedes (8) and other medical calculators (12).

**Table 2:** Doctors' perceptions of usefulness of various functions of the personal digital assistant (PDA)

Function	Useful*	Owns software applications^†^
Drug information	45 (83.3)	16 (29.6)
Medical references	44 (81.5)	27 (50.0)
Scheduling/calendar	38 (70.4)	Not applicable
Medical calculators	37 (68.5)	18 (33.3)
Documentation	17 (31.5)	Not applicable

* Data are presented as the number (%) of doctors who chose "Strongly agree" or "Agree", as opposed to "Not sure", "Disagree" or "Strongly disagree" when the 54 doctors who owned personal digital assistants were asked if each function was useful for acquiring medical knowledge.

^† ^Data are presented as the number (%) of doctors who owned the relevant medical software applications.

As for potential drawbacks of the PDA, the perceptions of all 134 respondents on these are listed in Table 3. The greatest concerns highlighted were the fear of loss and breakage, and the preference for working with desktop computers and paper. Only a small minority felt that the PDA made one look unprofessional, was too technical, or worked too slowly.

**Table 3:** Doctors' perceptions of potential drawbacks of the personal digital assistant (PDA)

Function	Agree*
Fear of loss and breakage	69 (51.5)
Prefer desktops	61 (45.5)
Prefer paper	57 (42.5)
Cumbersome to carry	49 (36.6)
Short battery life	45 (33.6)
Limited memory	42 (31.3)
Difficult data entry	41 (30.6)
Fear of over-reliance	39 (29.1)
Screen too small	30 (22.4)
Looks unprofessional	22 (16.4)
Too technical	19 (14.2)
Works too slowly	17 (12.7)

* Data are presented as the number (%) of doctors who chose "Strongly agree" or "Agree", as opposed to "Not sure", "Disagree" or "Strongly disagree" when all 134 doctors were asked if they agreed with each statement about the potential drawbacks of the personal digital assistant.

Of the remaining 80 doctors who did not own a PDA, only 14 (17.5%) thought they would buy one in the next 1 year (Figure 1).

### Use of UpToDate

**Figure 2:**
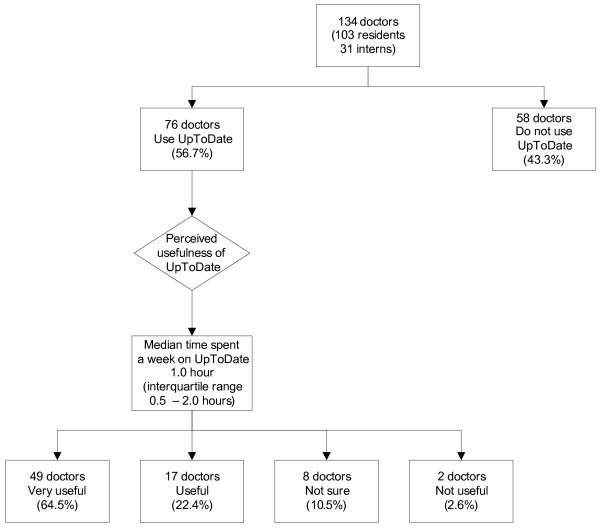
Doctors' use and perception of UpToDate.

As depicted in Figure 2, 76 doctors (58 residents and 18 interns) had previously used UpToDate. The median time spent per week was 1.0 hour (IQR 0.5 – 2.0 hours). The median frequency of use per week was 3.0 times (IQR 2.0 – 5.0 times). The median score on a Likert scale of 1 to 5 for the perceived usefulness of UpToDate was 1.0 (IQR 1.0 – 1.0). The median time taken to find an answer per search was 5.0 minutes (IQR 3.0 – 10.0 minutes).

Most of these 76 doctors felt that hospitals should have institutional subscriptions to UpToDate, and most would recommend UpToDate to a colleague (Table 4). However, most would not subscribe personally to UpToDate even if the hospital ceased its institutional subscription. They were also less enthusiastic in pronouncing that UpToDate had led to a change in their diagnoses or management of patients, or that UpToDate helped to avoid referrals to other specialties (Table 4). Among the 58 residents who used UpToDate, about half had used it in their clinics. Indeed, among the users of UpToDate, residents used it more frequently than interns (median 3.0 times per week [IQR 2.0–5.3 times] versus 1.5 times [IQR 1.0–3.5 times], *p *= 0.004, Mann-Whitney U test). Table 4 also shows the features of UpToDate which the doctors felt made it popular.

**Table 4:** Features of UpToDate which make it popular

Feature	Agree*
Subscriptions and recommendations	
Hospitals should subscribe	72 (94.7)
Will subscribe personally if hospital does not	24 (31.6)
Will recommend to a colleague	71 (93.4)
	
Use of UpToDate	
Has led to a change of management	44 (57.9)
Has led to a change of diagnosis	28 (36.8)
Helps avoid referrals to other specialties	33 (43.4)
Used in clinics^†^	30 (51.7)
	
Features of UpToDate which make it popular	
Synthesis of relevant information	74 (97.4)
Updated regularly	71 (93.4)
Comprehensive references	69 (90.8)
Wide collection of subspecialties	70 (92.1)
Easy-to-use recommendations	70 (92.1)

* Data are presented as the number (%) of doctors who chose "Strongly agree" or "Agree", as opposed to "Not sure", "Disagree" or "Strongly disagree" when the 76 doctors who had used UpToDate were asked if they agreed with each statement about UpToDate. sagree" or "Strongly disagree".

^† ^Only the answers of the 58 residents who had used UpToDate are shown; the 18 interns who had used UpToDate are excluded as interns did not run clinics.

Only 93 doctors (69.4%) were aware that our hospital had an institutional subscription to UpToDate. Of these, 4 doctors (3.0%) had a personal subscription to UpToDate. Trainees in the fields of internal medicine, paediatrics, obstetrics and gynaecology, and family medicine – specialties which are featured in UpToDate – were more likely to be aware that the hospital had an institutional subscription to UpToDate (90.0% versus 65.8%, p = 0.03, chi-square test), more likely to have used UpToDate previously (85.0% versus 51.8%, p = 0.006, chi-square test), more likely to find UpToDate useful (70.0% versus 45.6%, p = 0.04, chi-square test), and spent more time using UpToDate (median 1.0 hour [IQR 0.4–2.0 hours] versus median 0.25 hours [IQR 0–1.0 hours], p = 0.009, Mann-Whitney U test) compared to other doctors.

### Users of both medical software applications on PDAs and UpToDate

There were 23 doctors (17.2%) who used both UpToDate and PDAs downloaded with medical software. Among them, although the same amount of time was spent per week on both tools, PDAs were used more frequently than UpToDate. Nevertheless, UpToDate was perceived to be more useful than the medical software applications downloaded on the 23 PDAs for acquiring medical knowledge (Table 5).

**Table 5:** Doctors' use of UpToDate and the personal digital assistant (PDA)

	Only 23 respondents who used both UpToDate and PDAs with medical software applications		
Characteristic	UpToDate	PDA	*p *value

Time spent per week*	1.0 hr (1.0 – 2.0)	1.0 hr (0.5 – 2.0)	0.21
Frequency of use per week*	3.0 times (2.0 – 5.0)	12.0 times (5.0 – 18.0)	< 0.001
Usefulness^†^	1.0 (1.0 – 1.0)	2.0 (1.0 – 2.0)	0.005

* Data are presented as median (interquartile range); comparisons made using the Wilcoxon signed rank test.

^† ^Data are presented as median score (interquartile range) on a Likert scale in which 1 = strongly agree, 2 = agree, 3 = not sure, 4 = disagree, 5 = strongly disagree, when doctors were asked if UpToDate and the PDA was useful for acquiring medical knowledge; comparison made using the Wilcoxon signed rank test.

## Discussion

As opposed to the findings of previous studies [1,14,15], in general, our residents and interns did not spend much time using either the PDA or UpToDate. Our study therefore provides a good opportunity to explore the reasons for the under-utilisation of these tools.

### Use of PDAs

Internationally, the popularity of the PDA is rapidly increasing among doctors [9,10]. Systematic reviews of multiple studies have found that between 45% and 85% of doctors use PDAs [14,15], and that physicians in training were more likely to use one than more experienced doctors [15]. In our study, 40.3% of our house staff owned a PDA, a rate which is obviously lower than that found in other studies. Even among the owners of PDAs, the median time spent using the PDA per week was only 0.5 hours. This is despite most owners of PDAs reporting use for both work and personal purposes. In addition, only a small minority of those who did not own a PDA would want to buy one in the next 1 year. Aside from the fear of breakage and loss – concerns which are prevalent among PDA users [16,17] – many doctors preferred working with desktops and paper, suggesting that the advantage of portability of PDAs was not sufficient to persuade most doctors to make the switch to using PDAs.

Further analysis of our results yields the necessary information to explain the relative under-use of PDAs by these doctors. Most PDA owners in our survey perceived that the PDA provided useful drug information, medical references, scheduling functions and medical calculators. These are the same features which have been highlighted in other medical studies on PDAs [14-16,18-24]. However, although these functions were rated highly, only half of our PDA owners had medical references downloaded onto their PDAs (interestingly, none of the owners had UpToDate downloaded on their PDAs [12,13]), and only one-third or less had medical calculators and drug information software applications downloaded. In previous studies, residents and medical students who were provided with PDAs which were already loaded with the relevant medical software applications usually rated their experience with the PDAs highly [25-27]. In all, this suggests that in order to make the PDA more effective as an information tool for house staff, residency programmes should strongly consider the provision of the necessary subscription for PDA medical software applications, along with technical support and the installation of these applications [24].

### Use of UpToDate

With regard to UpToDate, more than 90% of all users in our study agreed that the following features made it popular: the synthesis of relevant information, being updated regularly, its comprehensive references, its wide collection of subspecialties, and its easy-to-use recommendations. Given that UpToDate features topics in internal medicine, paediatrics, obstetrics and gynaecology and family medicine, it was understandably more popular among trainees within these disciplines. Indeed, prior studies that had demonstrated the popularity of UpToDate had mostly evaluated doctors or medical students within these disciplines [1,11,16,28,29].

Our study however revealed two concerns on UpToDate. First, although UpToDate was generally perceived to be useful, only slightly more than half of the house staff had used UpToDate. Although our institutional subscription began 5 months before the study, 30.6% of residents and interns did not know about it. This implies that more work needs to be done to inform our doctors of the resources available in the hospital. Second, although 86.8% of UpToDate users found the resource useful, only slightly more than half of them felt that it had led to a change of management in their practice, and only slightly less than half of them felt that it had led to a change of diagnosis and a decrease in the amount of referrals to other specialties. This may reflect a lack of confidence or ability to integrate new information into actual day-to-day medicine. Senior doctors should be mindful of this and build a working environment which encourages learning and the adoption of new clinical practices [3,30,31].

### Users of both medical software applications on PDAs and UpToDate

The use patterns of the 23 doctors who have used both medical software applications on PDAs and UpToDate nicely sum up some of the features of these 2 tools. Although PDAs were used more frequently, more time was spent on UpToDate. This highlights the portable nature and accessibility of PDAs [9,10] – which may be used for quick information retrieval by busy house staff – as opposed to UpToDate, which provides textbook-like information, usually on desktop computers, at a more leisurely pace [12]. It must be emphasised that our survey did not compare the usefulness of UpToDate versus PDAs per se. Rather, we found that the medical software applications downloaded on our doctors' PDAs was perceived to be less useful than UpToDate for retrieving medical information. This may again reflect a lack of technical support for doctors using PDAs.

### Limitations

The limitations of our study should be acknowledged. First, since the study was based in a tertiary care hospital in Singapore, the results may not be generalisable to community-based hospitals and residency training programmes, and to the developing world. Second, though the response rate to our questionnaire was reasonably good at 79.8%, we do not have sufficient information on the non-respondents to determine the presence of a response bias. Third, as this is a questionnaire study, the validity of our results depends on how the respondents' answers truly reflected their actual practice. Also, the questions in the survey were generated by us and have not previously been validated. Fourth, we limited the questionnaire to the assessment of the use of PDAs to acquire medical knowledge and did not evaluate the integration of PDAs with administrative work processes and clinical information systems in the hospital [9,10,14,15]. Fifth, while we directly compared the time spent and perceived usefulness of PDAs and UpToDate, given their very different functions, our questionnaire could not provide a direct comparison of their various pros and cons. Sixth, various advances in both tools have been made since the time of our survey. For example, more topics are now covered by UpToDate, and refinements have been made to its search engine. Meanwhile, the distinction between PDAs and phones is being increasingly blurred with the rapid emergence of smart phones with PDA functionality [9,10]. Importantly, although none of our house staff's PDAs contained software for UpToDate, at the time of our survey, UpToDate could actually be downloaded onto PDAs with a Windows CE system but not a Palm operating system (nevertheless, Now, not only can UpToDate may be downloaded onto most PDAs, faster wireless access has also facilitated the use of UpToDate on PDAs and smart phones with web browsing capabilities.

## Conclusion

To conclude, although UpToDate and various PDA software applications, including those for drug information, medical references, scheduling and medical calculators, were deemed useful by some of the residents and interns in our study, both digital tools were under-utilised. More should be done to facilitate the use of medical software applications on PDAs, to promote awareness of tools for evidence-based medicine such as UpToDate, and to facilitate the application of evidence-based medicine in daily clinical practice.

## List of abbreviations

IQR: interquartile range; PDA: personal digital assistant.

## Competing interests

The authors declare that they have no competing interests.

## Authors' contributions

JP conceived of and coordinated the study, performed the statistical analysis and drafted the manuscript. TKL participated in its design and helped to draft the manuscript. Both authors read and approved the final manuscript.

## Pre-publication history

The pre-publication history for this paper can be accessed here:



## Supplementary Material

Additional file 1 Questionnaire on the use of information technology in routine clinical practice. Click here for file
