# Supplementary material for: How residents and interns utilise and perceive the personal digital assistant and UpToDate
Source: BMC Med Educ. 2008 Jul 14;8:39. doi: 10.1186/1472-6920-8-39 (PMC2483706; doi:10.1186/1472-6920-8-39)
Supplement: Additional file 1 — Questionnaire on the use of information technology in routine clinical practice. [file 1472-6920-8-39-S1.pdf]

Questionnaire on the use of information technology
in routine clinical practice

NAME: ____

DATE: ____

Dear Colleague,

Thank you for taking the time to complete this questionnaire. This is a survey to find out how much local doctors rely on information technology to acquire medical knowledge in routine clinical practice.

Please be reassured that you WILL remain anonymous. Please be FRANK in your answers and tell us what you TRULY feel. There is NO need to be politically correct. This survey is NOT intended to assess you. Thank you once again.

Regards,

Dr Phua Jason
Registrar
Division of Respiratory and Critical Care Medicine
Department of Medicine
National University Hospital

Personal data

Sex: ___________________________________

Age: ___________________________________

Year of graduation: _______________________

Current post: House Officer / Medical Officer* (circle accordingly)

Current status: Non-trainee / Basic Specialty Trainee* (circle accordingly)

If BST, state field (including GP trainee): ____________________________________

Current division / department / hospital: _____________________________________

Please estimate the average duration of time you spend a week on the following:

The Personal Digital Assistant (PDA) to retrieve medical information	__________ hours	__________ mins
Uptodate.com	__________ hours	__________ mins
Medline (Pubmed)	__________ hours	__________ mins
Cochrane database (including Cochrane abstracts)	__________ hours	__________ mins
Original research papers online	__________ hours	__________ mins
Original research papers from actual hard copy journals	__________ hours	__________ mins
Review articles online	__________ hours	__________ mins
Review articles from actual hard copy journals	__________ hours	__________ mins
MD Consult	__________ hours	__________ mins
Medscape	__________ hours	__________ mins
Postgraduate Medicine Online	__________ hours	__________ mins
Medreviews.com	__________ hours	__________ mins
Other sources from the internet to retrieve other medical information	__________ hours	__________ mins
>>> Please specify what source(s): ______________________	__________ hours	__________ mins
______________________	__________ hours	__________ mins
______________________	__________ hours	__________ mins
Hard copy medical textbooks	__________ hours	__________ mins
Online medical textbooks, e.g. Harrison's online	__________ hours	__________ mins
Teaching rounds / sessions / grand rounds / journal clubs	__________ hours	__________ mins

Please tick the appropriate boxes according to how you feel about the following:

The following are useful to YOU for acquiring medical knowledge:

	Strongly agree	Agree	Not sure	Disagree	Strongly disagree
The Personal Digital Assistant (PDA) to acquire medical knowledge	☐	☐	☐	☐	☐
Uptodate.com	☐	☐	☐	☐	☐
Medline (Pubmed)	☐	☐	☐	☐	☐
Cochrane database (including Cochrane abstracts)	☐	☐	☐	☐	☐
Original research papers online	☐	☐	☐	☐	☐
Original research papers from actual hard copy journals	☐	☐	☐	☐	☐
Review articles online	☐	☐	☐	☐	☐
Review articles from actual hard copy journals	☐	☐	☐	☐	☐
MD Consult	☐	☐	☐	☐	☐
Medscape	☐	☐	☐	☐	☐
Postgraduate Medicine Online	☐	☐	☐	☐	☐
Medreviews.com	☐	☐	☐	☐	☐
Other sources from the internet to acquire other medical knowledge	☐	☐	☐	☐	☐
>>> Please specify what source(s): ________________________	☐	☐	☐	☐	☐
________________________	☐	☐	☐	☐	☐
________________________	☐	☐	☐	☐	☐
Hard copy medical textbooks	☐	☐	☐	☐	☐
Online medical textbooks, e.g. Harrison's online	☐	☐	☐	☐	☐
Teaching rounds or sessions	☐	☐	☐	☐	☐

The Personal Digital Assistant (PDA) / handheld computer

Your own PDA / handheld computer

Do you own your own PDA / handheld computer? Yes / No* (circle accordingly)

>>> If yes, state brand and version: ________________________________

How long have you used a PDA? ______________________________

For what purpose do you use your PDA? Mainly work-related / Mainly personal / Both* (circle accordingly)

>>> If no, do you think you will buy one in the next 1 year?

Definitely yes / Yes / Not sure / No / Definitely no* (circle accordingly)

PDA software in general

If you are using a PDA now, on average, how many times do you use it to retrieve any kind of medical information, including electronic medical references / textbooks, drug information and medical calculators?
(Include every time you switch on the PDA to check on a particular topic, e.g. drug dose)

______________________ times per week

Specific PDA software

List the PDA software that you own:

**Medical references / textbooks:**	**No. of times used per week**	**Useful to YOU for acquiring medical knowledge?**				
		Strongly agree	Agree	Not sure	Disagree	Strongly disagree
1. ______________________	________ per wk	☐	☐	☐	☐	☐
2. ______________________	________ per wk	☐	☐	☐	☐	☐
3. ______________________	________ per wk	☐	☐	☐	☐	☐
4. ______________________	________ per wk	☐	☐	☐	☐	☐
5. ______________________	________ per wk	☐	☐	☐	☐	☐
6. ______________________	________ per wk	☐	☐	☐	☐	☐
7. ______________________	________ per wk	☐	☐	☐	☐	☐
8. ______________________	________ per wk	☐	☐	☐	☐	☐
9. ______________________	________ per wk	☐	☐	☐	☐	☐
10. ______________________	________ per wk	☐	☐	☐	☐	☐
11. ______________________	________ per wk	☐	☐	☐	☐	☐
12. ______________________	________ per wk	☐	☐	☐	☐	☐

**Drug information:**	**No. of times used per week**	**Useful to YOU for acquiring medical knowledge?**				
		Strongly agree	Agree	Not sure	Disagree	Strongly disagree
1. ______________________	________ per wk	☐	☐	☐	☐	☐
2. ______________________	________ per wk	☐	☐	☐	☐	☐
3. ______________________	________ per wk	☐	☐	☐	☐	☐
4. ______________________	________ per wk	☐	☐	☐	☐	☐
5. ______________________	________ per wk	☐	☐	☐	☐	☐

**Medical calculators:**	**No. of times used per week**	**Useful to YOU for acquiring medical knowledge?**				
		Strongly agree	Agree	Not sure	Disagree	Strongly disagree
1. ______________________	________ per wk	☐	☐	☐	☐	☐
2. ______________________	________ per wk	☐	☐	☐	☐	☐
3. ______________________	________ per wk	☐	☐	☐	☐	☐
4. ______________________	________ per wk	☐	☐	☐	☐	☐
5. ______________________	________ per wk	☐	☐	☐	☐	☐

PDA functions

Please tick the appropriate boxes according to how you feel about the following statements regarding the PDA.

Please tick the boxes regardless of whether or not you are using a PDA / handheld computer now.

	Strongly agree	Agree	Not sure	Disagree	Strongly disagree
It is too cumbersome to carry around.	☐	☐	☐	☐	☐
There is limited memory.	☐	☐	☐	☐	☐
The battery life is too short.	☐	☐	☐	☐	☐
The device works too slowly.	☐	☐	☐	☐	☐
Data entry using graffiti is too difficult.	☐	☐	☐	☐	☐
The screen is too small and the words are difficult to read.	☐	☐	☐	☐	☐
It is too technical and therefore too difficult to use.	☐	☐	☐	☐	☐
I prefer working with paper.	☐	☐	☐	☐	☐
I prefer working with desktop computers.	☐	☐	☐	☐	☐
There is always a fear of loss and breakage.	☐	☐	☐	☐	☐
One may become too over-reliant on it.	☐	☐	☐	☐	☐
Using the device in front of patients looks unprofessional.	☐	☐	☐	☐	☐

The following functions are useful to YOU for acquiring medical knowledge: (tick NA if you are not using a PDA)

	Strongly agree	Agree	Not sure	Disagree	Strongly disagree	NA: No PDA
Scheduling / calendar	☐	☐	☐	☐	☐	☐
Documentation / billing	☐	☐	☐	☐	☐	☐
Drug information	☐	☐	☐	☐	☐	☐
Medical references	☐	☐	☐	☐	☐	☐
Medical calculators	☐	☐	☐	☐	☐	☐

UpToDate

Does your institution subcscribe to the online medical resource UpToDate?	Yes / No* (circle accordingly)
Do you personally subcscribe to the online medical resource UpToDate?	Yes / No* (circle accordingly)
On average, how many times a week do you use UpToDate?	___________ times per week
On average, how long does it take to find an answer on UpToDate? (enter NA if you have never used UpToDate)	___________ mins

Please tick the appropriate boxes according to how you feel about the following:
(tick NA if you have never used UpToDate)

	Strongly agree	Agree	Not sure	Disagree	Strongly disagree	NA
UpToDate helps to avoid referrals to other specialties.	☐	☐	☐	☐	☐	☐
UpToDate is faster than other means of finding information.	☐	☐	☐	☐	☐	☐
Using UpToDate has led to a change of diagnosis in my clinical practice.	☐	☐	☐	☐	☐	☐
Using UpToDate has led to a change of patient management in my clinical practice.	☐	☐	☐	☐	☐	☐
UpToDate helps make me comfortable with my decisions.	☐	☐	☐	☐	☐	☐
UpToDate helps me to provide the best care for my patients.	☐	☐	☐	☐	☐	☐
UpToDate makes me a better doctor.	☐	☐	☐	☐	☐	☐
I use UpToDate in my clinics.	☐	☐	☐	☐	☐	☐
**The following are features of UpToDate which make it popular:**						
Updated regularly	☐	☐	☐	☐	☐	☐
Synthesis of relevant information	☐	☐	☐	☐	☐	☐
Easy-to-use recommendations	☐	☐	☐	☐	☐	☐
Wide collection of subspecialties	☐	☐	☐	☐	☐	☐
Comprehensive references	☐	☐	☐	☐	☐	☐
**Subscription and recommendations**						
Hospitals should have institutional subscriptions to UpToDate.	☐	☐	☐	☐	☐	☐
Do you think you will subscribe to UpToDate in the next one year if your institution does not or no longer subscribes?	☐	☐	☐	☐	☐	☐
Do you think you will subscribe to UpToDate in the next one year if your institution subscribes?	☐	☐	☐	☐	☐	☐
I would recommend UpToDate to a colleague.	☐	☐	☐	☐	☐	☐
